# The Effect of *Trim5* Polymorphisms on the Clinical Course of HIV-1 Infection

**DOI:** 10.1371/journal.ppat.0040018

**Published:** 2008-02-01

**Authors:** Daniëlle van Manen, Maarten A. N Rits, Corrine Beugeling, Karel van Dort, Hanneke Schuitemaker, Neeltje A Kootstra

**Affiliations:** 1 Department of Clinical Viro Immunology, Sanquin Research, Landsteiner Laboratory, University of Amsterdam, Amsterdam, The Netherlands; 2 Center of Infection and Immunity Amsterdam (CINIMA), University of Amsterdam, Amsterdam, The Netherlands; 3 Department of Experimental Immunology, Amsterdam Medical Center, Amsterdam, The Netherlands; Institute for Research in Biomedicine, Switzerland

## Abstract

The antiviral factor tripartite interaction motif 5α (Trim5α) restricts a broad range of retroviruses in a species-specific manner. Although human Trim5α is unable to block HIV-1 infection in human cells, a modest inhibition of HIV-1 replication has been reported. Recently two polymorphisms in the *Trim5* gene (H43Y and R136Q) were shown to affect the antiviral activity of Trim5α *in vitro*. In this study, participants of the Amsterdam Cohort studies were screened for polymorphisms at amino acid residue 43 and 136 of the *Trim5* gene, and the potential effects of these polymorphisms on the clinical course of HIV-1 infection were analyzed. In agreement with the reported decreased antiviral activity of Trim5α that contains a Y at amino acid residue 43 *in vitro*, an accelerated disease progression was observed for individuals who were homozygous for the 43Y genotype as compared to individuals who were heterozygous or homozygous for the 43H genotype. A protective effect of the 136Q genotype was observed but only after the emergence of CXCR4-using (X4) HIV-1 variants and when a viral load of 10^4.5^ copies per ml plasma was used as an endpoint in survival analysis. Interestingly, naive CD4 T cells, which are selectively targeted by X4 HIV-1, revealed a significantly higher expression of Trim5α than memory CD4 T cells. In addition, we observed that the 136Q allele in combination with the −2GG genotype in the 5′UTR was associated with an accelerated disease progression. Thus, polymorphisms in the *Trim5* gene may influence the clinical course of HIV-1 infection also underscoring the antiviral effect of Trim5α on HIV-1 *in vivo*.

## Author Summary

The clinical course of HIV-1 infection is highly variable between individuals, and host genetic variations may at least account for part of these differences. Recently two single nucleotide polymorphisms in the *tripartite interaction motif 5* gene (*Trim5*) have been reported to affect the antiviral activity of the Trim5α protein. Here we analyzed the effect of these polymorphisms on the clinical course of HIV-1 infection in participants of the Amsterdam Cohort studies. We observed an accelerated disease progression for individuals who were homozygous for the 43Y genotype that has been associated with a decreased antiviral activity of Trim5α *in vitro*. The 136Q genotype has *in vitro* been associated with a slightly higher anti-HIV-1 activity. We observed a protective effect of the 136Q genotype only after the emergence of CXCR4-using HIV-1 variants using viral load above 10^4.5^ copies per ml plasma as an endpoint in survival analysis. These results suggest that genetic variations in the *Trim5* gene may influence the clinical course of HIV-1 infection and confirm a role of Trim5α on HIV-1 *in vivo*.

## Introduction

The susceptibility to HIV-1 infection and subsequent disease progression is highly variable between individuals. Host genetic variations have previously been demonstrated to account for at least part of these differences. Polymorphisms in chemokine receptors that serve as HIV-1 coreceptors, or in their natural ligands, have been associated with reduced susceptibility to infection as well as disease progression [1–4]. Furthermore, certain HLA types have been correlated with the clinical course of infection [5–8].

Variations in genes involved in innate immunity may also contribute to the differential susceptibility of humans to HIV-1 infection and the highly variable outcome of the disease. Recently, the tripartite interaction motif 5α (Trim5α) has been identified as part of the intrinsic immunity that protects human and non-human primates against retroviral infection [9,10]. Trim5α targets the capsid of the incoming retrovirus in the cytoplasm directly after entry and interferes with viral replication at an early post-entry step most likely at the poorly understood uncoating process [11–19]. Species-specific variations in Trim5α account for the restriction pattern of specific retroviruses [20–26]. For example, HIV-1 replication is blocked efficiently by Trim5α of rhesus macaques and African green monkeys, whereas SIV-mac is only restricted by Trim5α from African green monkey. Human Trim5α efficiently blocks N-tropic MLV and equine infectious anaemia virus, but is much less efficient in restricting HIV-1 replication. This indicates that HIV-1 has at least partially adapted to the human variant of this restriction factor. Recently, we observed that Trim5α escape variants developed late in infection in a proportion of the HIV-1 infected individuals [27]. The emergence of the escape variants was preceded by a prolonged asymptomatic phase, indicating that Trim5α mediated suppression of viral replication indeed plays a role in HIV-1 pathogenesis.

The potential role of polymorphisms within the *Trim5* gene on HIV-1 susceptibility has recently been studied [28–31]. Of the eight nonsynonymous polymorphisms that have been identified in the *Trim5* gene, two have been reported to have functional consequences with regard to the antiviral activity of Trim5α (H43Y and R136Q) [28,30]. The H43Y is located in the RING domain of Trim5α and may impair its putative E3 ligase activity [28,30]. Indeed, the 43Y variant of Trim5α was less efficient in restricting HIV-1 replication *in vitro* [28,30]. The R136Q polymorphism has been associated with a slightly higher anti-HIV-1 activity of Trim5α [30]. In agreement, the R136Q polymorphism was more frequently observed in high risk seronegative as compared to HIV-1 infected individuals in a cohort of African Americans [30], although not confirmed in other study populations [29,30]. So far no significant association between *Trim5* polymorphisms and HIV-1 disease progression have been demonstrated [29,31,32].

Here we studied the effect of the Trim5α H43Y and R136Q polymorphisms on the clinical course of HIV-1 infection in participants of the Amsterdam Cohort studies. In addition, we analyzed whether the R136Q genotype in combination with a SNP in the 5′UTR of *Trim5* (−2G/C) was associated with susceptibility to HIV-1 infection or disease progression.

## Results

### Distribution of H43Y and R136Q *Trim5* Genotypes

The prevalence of Trim5α polymorphisms H43Y and R136Q was studied in 327 HIV-1 positive participants of the Amsterdam Cohort studies. For the H43Y polymorphism a minor allele frequency of 0.115 was observed. Of the 327 HIV-1 positive participants, 61 (18.7%) were heterozygous and 7 (2.1%) were homozygous for the 43Y allele. The R136Q polymorphism was observed at a minor allele frequency of 0.379. Of the 327 participants, 156 (47.7%) were heterozygous and 46 (14.1%) were homozygous for the 136Q allele. Six mutually exclusive haplotypes were observed for the combination of R136Q and H43Y polymorphisms: 43HH/136RR (n = 88), 43HH/136RQ (n = 125), 43HH/136QQ (n = 46), 43HY/136RQ (n = 31), 43HY/136RR (n = 30) and 43YY/136RR (n = 7). The 43Y polymorphism was not observed in the group homozygous for the 136QQ genotype and the 136Q polymorphism was never observed in combination with a homozygous 43YY genotype, also confirming that the 43Y polymorphism and the 136Q polymorphism are not located on the same allele [29,31]. No significant differences in the H43Y or R136Q minor allele frequencies were observed between the HIV-1 seropositive individuals and healthy controls (allele frequencies of 0.106 and 0.389 for H43Y and R136Q, respectively).

### Effect of the H43Y *Trim5* Genotype on the Clinical Course of HIV-1 Infection

**Figure 1:**
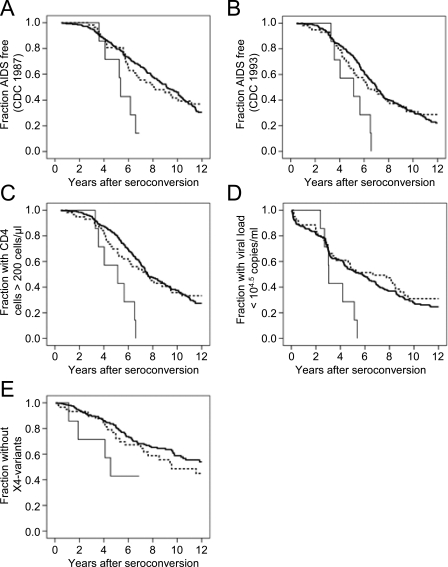
Survival Analysis for the H43Y Genotype Kaplan Meier analysis for time in years from seroconversion to AIDS according to the CDC 1987 definition (A), to AIDS according to the CDC 1993 definition (B), to CD4 count below 200 cells/μl blood (C), to viral RNA load above 10^4.5^ copies per ml plasma (D), and to first detection of X4-variants (E) based on the H43Y genotype. Bold lines indicate individuals with the wild type genotype (43HH); dashed black lines indicate individuals heterozygous for the 43Y genotype (43HY); thin black lines indicate individuals homozygous for the 43Y genotype (43YY).

Kaplan Meier and Cox Proportional Hazard analysis with clinical AIDS (Definition CDC 1987 and 1993), CD4 T cell counts below 200 cells/μl blood, and plasma viral RNA load above 10^4.5^ copies per ml plasma were used as end points to determine the effect of polymorphisms in the *Trim5* gene on disease progression. We observed an accelerated disease progression in the group homozygous for the 43Y allele relative to the 43 HH wild type genotype, with a relative hazard (RH) of 3.1 (*p* = 0.006) and 2.8 (*p* = 0.007) for AIDS diagnosis according to the 1987 or 1993 CDC definition, respectively (Figure 1A and 1B; Table 1). An accelerated progression rate was also observed when CD4 T cell counts below 200 cells per μl blood were used as end point (Figure 1C; Table 1). The median viral RNA load of participants of the Amsterdam cohort progressing to AIDS has previously been determined at 10^4.5^ copies HIV-1 RNA per ml plasma [1]. When viral RNA load above 10^4.5^ copies per ml plasma was used as endpoint in the survival analysis, we again observed an accelerated disease progression for individuals homozygous for the 43Y genotype (Figure 1D; Table 1). The heterozygous genotype (43HY) was not associated with delayed disease progression (Figure 1).

**Table 1:**
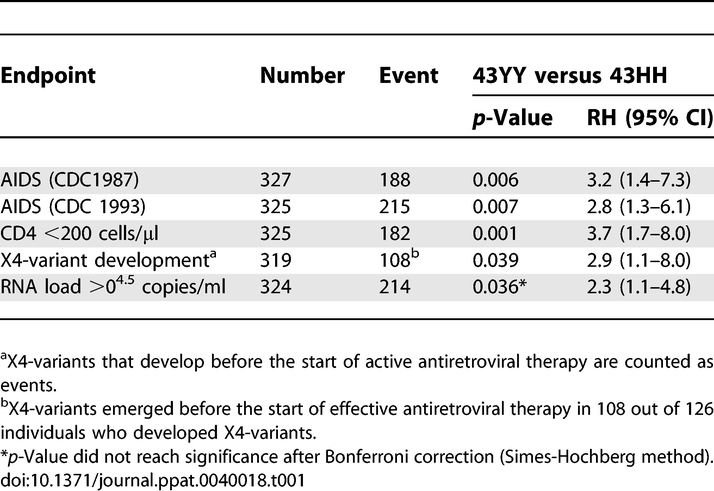
Cox Regression Analysis for Progression to AIDS, CD4 Cells <200 Cells/μl, and X4-Variant Development

Development of CXCR4 using HIV-1 variants (X4-variants) has previously been associated with an accelerated disease progression [33]. The ability of HIV-1 variants to use CXCR4 as a coreceptor and replicate in MT2 cells was analyzed routinely during follow-up in the cohort studies in 319 of 327 individuals from our study population. During the course of infection X4-variants developed in 126 individuals. No association between the prevalence of X4-variants and the H43Y genotype could be observed (data not shown). However, X4-variants did develop more rapidly in individuals who were homozygous for the 43Y genotype (Figure 1E; Table 1).

Our study population consisted of 130 participants who seroconverted for HIV-1 antibodies during follow-up and 197 seroprevalent participants with an imputed seroconversion date [34]. Inclusion of seroprevalent participants in our analysis did not bias our data and Cox regression analysis stratifying for seroconvertors and seroprevalent participants gave similar results (data not shown).

### Effect of the R136Q *Trim5* Genotype on the Clinical Course of HIV-1 Infection

Next we examined a potential role for the R136Q polymorphism in *Trim5* on the clinical course of HIV-1 infection. Using clinical AIDS (Definition CDC 1987 and 1993), CD4 T cell counts below 200 cells/μl blood or viral RNA load above 10^4.5^ copies per ml plasma as endpoint in Kaplan Meier and Cox proportional hazard analysis, no significant associations between the 136RQ or 136QQ genotype and the clinical course of infection were revealed (data not shown). The R136Q polymorphism also had no effect on the time to first detection of X4-variants (data not shown). In addition, the prevalence of CXCR4 using HIV-1 variants was not associated with the R136Q genotype.

**Figure 2:**
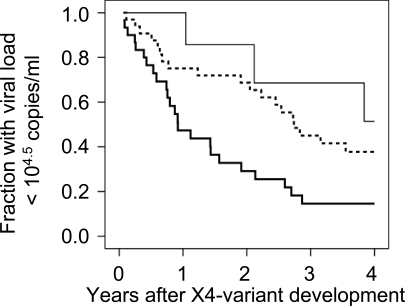
Survival Analysis for R136Q Genotype after Emergence of X4-Variants Kaplan Meier analysis for time in years from the moment of first detection of X4-variants to viral RNA load above 10^4.5^ copies per ml plasma based on the R136Q genotype. Bold black lines indicate individuals with the wild type genotype (136RR); dashed black lines indicate individuals heterozygous for the 136Q genotype (136RQ); thin black lines indicate individuals homozygous for the 136Q genotype (136QQ).

Next we analyzed whether a potential effect of the R136Q *Trim5* genotype was dependent on the coreceptor usage of the virus present. The R136Q genotype had no significant effect on the clinical course of infection when only CCR5 using HIV-1 variants (R5-variants) were present irrespective the end point used in the survival analysis (data not shown). However, a significant protective effect on disease progression associated with the R136Q genotype was observed after X4-variant development using the median viral RNA load of participants of the Amsterdam cohort progressing to AIDS (10^4.5^ copies per ml) as an end point in survival analysis, with a RH of 0.44 (*p* = 0.008) and 0.26 (*p* = 0.030) for the heterozygous 136RQ and homozygous 136QQ genotype, respectively as compared to the 136RR genotype (Figure 2; Table 2). At the moment of X4-development the viral load of individuals who were homozygous (QQ), heterozygous (RQ) or wild type (RR) for the amino acid residue at position 136 was similar (data not shown). The R136Q polymorphism was not associated with disease progression after X4-variant development using clinical AIDS or CD4 cell counts below 200 cells μl blood as end points (Table 2).

**Table 2:**
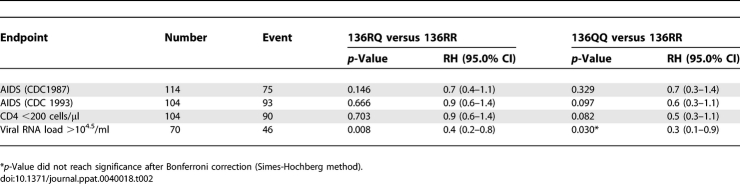
Cox Regression Analysis for Progression to AIDS, CD4 Cells <200 Cells/μl, or Viral RNA Load >10^4.5^/ml after X4-Variant Development

### Trim5α mRNA Expression Levels in Naïve and Memory CD4 T Cells

**Figure 3:**
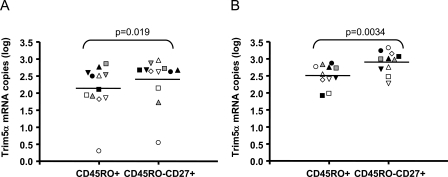
Analysis of Trim5α mRNA Levels in Naïve and Memory CD4 T Cells (A) Trim5α mRNA levels in naïve CD4 T cells (CD45RO-CD27+) and memory CD4 T cells (CD45RO+) obtained from healthy controls. (B) Average Trim5α mRNA levels in naïve and memory CD4 T cells during the course of infection from HIV-1 infected individuals. Trim5α mRNA levels are normalized for β-actin mRNA levels. Different symbols represent Trim5α mRNA levels of naïve and memory CD4 T cells from the different individuals.

The protective effect of the 136Q Trim5α variant on disease progression only after emergence of X4 variants may imply that Trim5α has a stronger effect on X4 variants than on R5 variants. Previously we demonstrated that R5 and X4 HIV-1 variants partially reside in different CD4 T cell compartments due to differential expression of coreceptors CCR5 and CXCR4 [35,36]. R5 variants were selectively isolated from CD4 memory T cells, whereas X4 variants were isolated from memory and naïve CD4 T cells. Here we analyzed whether differences of the Trim5α mRNA levels in naïve and memory CD4 T cell populations could contribute to the differential effect of the 136Q variant on X4 and R5 variants *in vivo*. Naïve and memory CD4 T cells were isolated from PBMC from 12 healthy controls by FACS sorting based on CD45RO and CD27 expression and Trim5α mRNA levels were analyzed by quantitative real time PCR. To correct for differences in input, Trim5α mRNA levels were normalized for β-actin mRNA levels. Trim5α mRNA levels were significantly higher in naïve (CD45RO−CD27+) CD4 T cells as compared to memory (CD45RO+) CD4 cells (*p* = 0.019) (Figure 3A).

In addition, Trim5α mRNA levels in naïve and memory CD4 T cells were analyzed in HIV-1 positive individuals early and late in the course of infection (23 PBMC samples from 11 individuals). Although no differences in Trim5α mRNA levels were observed during the course of infection, we again observed a significantly higher Trim5α mRNA level in naïve CD4 T cells as compared to memory CD4 T cells (*p* = 0.003) (Figure 3B).

### Effect of Combined R136Q and −2G/C Genotypes on Susceptibility to HIV-1 Infection and Disease Progression

Recently a G to C polymorphism at position −2 in the 5′UTR of the *Trim5* gene (−2G/C; rs3824949) in combination with the R136Q polymorphism has been associated with enhanced susceptibility to HIV-1 infection (136Q/−2G haplotype) and accelerated disease progression (136R/−2G haplotype) [29]. To analyze whether the combined R136Q and −2G/C genotype was also associated with HIV-1 susceptibility or disease progression in our study population, we genotyped our study population for the G to C polymorphism at position −2 (−2G/C). The −2C allele frequency was 0.486 and 0.418 in our HIV-1 positive individuals and healthy controls, respectively. When the −2G/C genotype was analyzed in combination with the R136Q genotype no significant differences in the distribution of the combined genotypes was observed between the HIV-1 infected individuals and the healthy controls. The −2GG genotype was not observed in combination with the homozygous 136Q genotype in both study populations.

**Figure 4:**
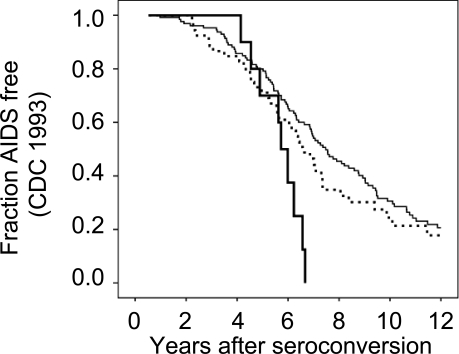
Survival Analysis for the −2G/C Genotype in Combination with the 136Q Genotype Kaplan Meier analysis for time in years from seroconversion to AIDS according to the CDC 1993 definition. Bold black lines indicate individuals with the −2GG genotype; dashed black lines indicate individuals with the −2GC genotype; thin black lines indicate individuals with the −2CC genotype.

Next we analyzed the effect of the combined R136Q and −2G/C genotypes on disease progression using clinical AIDS (Definition CDC 1993) as an end point. Individuals carrying the 136Q allele (136RQ and 136QQ) in combination with the −2GG genotype showed an accelerated progression to disease in comparison to the −2GC (*p* = 0.009; RH 2.6; 95% CI 1.3–5.2) and −2CC genotype (*p* = 0.056; RH 2.1; 95% CI 1.0–4.3) (Figure 4). In contrast to the study by Speelmon et al. [29], no significant effect of the −2G/C genotype on disease progression was observed in the group with the 136RR genotype.

### AIDS Incidence in Relation to H43Y Genotype and Other Progression Markers

Uni- and multivariate relative hazard analysis were used to determine the predictive value of the *Trim5* H43Y genotype (43YY) in combination with previously established prediction markers such as CD4 T cell count, plasma viral RNA load, the presence of X4-variants, and *CCR5*-genotype [1]. Univariate analysis indicated that homozygosity for the H43Y polymorphism, CD4 T cell counts below 500 cells per μl, viral RNA load above 10^4.5^ copies per ml plasma and the presence of X4-variants at 18–30 months after seroconversion were predictive for more rapid progression to AIDS, whereas heterozygosity for the *CCR5-*Δ*32* genotype had a protective effect (Table 3). Multivariate analysis at 2 years after seroconversion indicated that CD4 T cell counts below 500 cells per μl blood, viral RNA load above 10^4.5^ copies per ml plasma, the presence of X4-variants and homozygosity for the *Trim5* H43Y genotype (43YY) were independent predictors for progression to AIDS (Table 3). In our study population the homozygous H43Y genotype was not observed in combination with a heterozygous *CCR5-*Δ*32* genotype excluding this parameter from the multivariate analysis.

**Table 3:**
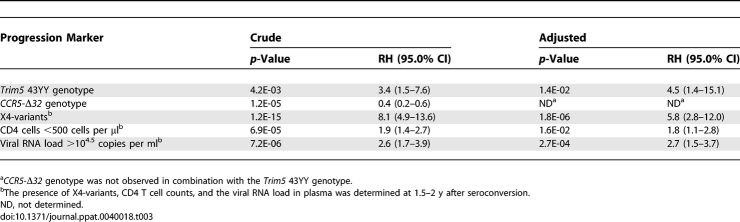
Univariate and Multivariate Relative Hazards for Progression to AIDS (CDC 1987) for the H43Y Genotype, Presence of X4-Variants, CD4 T Cell Count, Viral RNA Load in Plasma, or *CCR5* Genotype at Two Years after Seroconversion

## Discussion

Old World monkey Trim5α very efficiently blocks HIV-1 infection at an early step in the viral replication cycle, immediately after cellular entry. Human Trim5α is also able to interfere with HIV-1 infection, albeit less efficiently. Although this may suggest that HIV-1 has at least partially adapted to human Trim5α, we have recently provided the first evidence that Trim5α might still play a role in HIV-1 pathogenesis [27]. We observed that HIV-1 variants containing a H87Q mutation in the cyclophilin A binding region of capsid, which has previously been associated with escape from Trim5α [11–16], developed during the late phase of infection in a proportion of the HIV-1 infected individuals [27]. The emergence of these Trim5α escape variants was preceded by a prolonged asymptomatic phase implying that Trim5α contributed to control of virus replication *in vivo* and concomitantly selected for Trim5α resistant variants.

Two genetic polymorphisms in the human *Trim5* gene have recently been described to affect the antiviral activity of Trim5α on HIV-1. The H43Y polymorphism has been associated with an impaired anti-HIV-1 activity of Trim5α *in vitro* [28,30,32]. In agreement, we here observed that a 43YY homozygous genotype was predictive for an accelerated progression to AIDS, independent of CD4 cell counts, viral RNA load in plasma, and HIV-1 coreceptor usage at 18–30 months after seroconversion.

In previous studies, the H43Y genotype had no effect on HIV-1 disease progression [29–32]. Speelmon et al. observed no significant difference in viral RNA load in the period from 100 days until 2 years post infection between individuals who were homozygous (YY), heterozygous (HY) or wild type (HH) for amino acid residue 43 [29]. In agreement, we also did not observe a difference in viral RNA load at 2 years after seroconversion between the H43Y genotypic groups (data not shown). However, the studies by Speelmon et al. and by Goldschmidt et al. did also not show significant differences in CD4 cell decline between the H43Y genotypic groups [29,31]. Due to the low minor allele frequency for H43Y in the study population by Speelmon et al., which had a size of only 90 individuals, the number of individuals homozygous for the 43Y genotype might have been to low to observe significant difference between the genotypes. The discrepancy between our results and those of Goldschmidt et al. may lie in their relatively short average follow-up time of 3.2 years as compared to the average follow-up of 7.9 years on patients in our study. Nakayama et al. observed similar frequencies of homozygous 43YY and heterozygous 43HY genotypes in progressors and LTNP in a study population of Japanese HIV-1 infected individuals [32]. In our Amsterdam cohort however, none of the individuals homozygous for the 43YY genotype had an asymptomatic follow up of 10 years or more. Javanbakht et al. observed no significant differences in progression to CD4 T cell counts below 200 cells per μl blood, AIDS defining events or AIDS related deaths associated with the different H43Y genotypes in two large cohorts of African Americans and European Americans [30]. In the African American population the frequency of individuals homozygous for the 43YY genotype is however very low which might account for the discrepancy with our data. However, the minor allele frequency for H43Y in the European American cohort is similar to the frequency in the Amsterdam cohort (0.114 and 0.115 respectively). Unfortunately, lack of details on their European American cohort and their analyses make it difficult to bring up potential explanations for the inconsistency in results.

The R136Q polymorphism has been associated with a slight increase of the anti-HIV-1 activity of Trim5α *in vitro* [30], although not confirmed by others [28,29,31]. In agreement with earlier reports [29,31], we did not observe an effect of the R136Q polymorphism on disease progression. A protective effect of the 136Q variant was only evident in the phase of infection when X4-variants were present, where a delayed rise in viral load above the median load during progression to disease (10^4.5^ copies per ml) was observed in individuals who were homozygous (QQ) or heterozygous (RQ) for the 136Q genotype as compared to individuals with the wild type genotype (RR).

The protective effect of the 136Q Trim5α variant on disease progression only after the emergence of X4-variants, may imply that Trim5α *in vivo* affects replication of X4-variants more efficiently than R5-variants. X4-variants develop in 50% of the HIV-1 infected individuals during the natural course of infection after which R5- and X4-variants coexist [35–37]. While co-existing R5- and X4-variants infect memory CD4 T cells that co-express CCR5 and CXCR4, X4-variants have the unique ability to additionally infect naive CD4 T cells that selectively express CXCR4 [35,36]. Here we observed that naïve CD4 T cells expressed higher levels of Trim5α as compared to memory CD4 T cells. It is tempting to speculate that high Trim5α expression levels in naive T cells in combination with a more potent antiviral activity associated with the 136Q polymorphism provide prolonged control of HIV-1 replication in carriers of X4-variants.

Recently a G to C polymorphism at position −2 in the 5′UTR of the *Trim5* gene (−2G/C; rs3824949) in combination with the R136Q polymorphism has been associated with HIV-1 susceptibility and disease progression [29]. Speelmon et al. observed an enrichment of the 136Q/−2G haplotype in the HIV-1 positive population [29], however we were unable to confirm this and observed an equal distribution of the −2G/C polymorphism in combination with the 136Q allele between the HIV-1 positive population and the control group. However, the 136Q allele in combination with the −2GG genotype was associated with accelerated disease progression in our study population. Speelmon et al. observed an association between a faster CD4 T cell decline and the 136R/−2G haplotype [29], but we were unable to confirm this. In our study population we observed a −2C allele frequency of 0.486 and 0.418 in the HIV-1 positive study population and the control group, respectively, which is similar to the frequencies in the European American population reported by Javanbakht et al. [30]. However the frequencies for the −2C allele frequencies in the study populations of Speelmon et al. were much lower (0.38 in exposed seronegatives and 0.31 in HIV-1 infected population) [29]. Therefore, it cannot be excluded that differences in the distribution of the −2G/C polymorphism in different study populations account for the observed discrepancies in results.

Our data confirm a role of Trim5α in the clinical course of infection. In addition, they show that different genetic variants in Trim5α are associated with a differential clinical course of infection. Overall, these results may encourage exploiting the possibility of using Trim5α or alike derivatives in antiviral strategies.

## Materials and Methods

### Study participants.

The study population, 364 Caucasian, homosexual men enrolled in the Amsterdam Cohort studies (ACS) on the natural history of HIV-1 infection between October 1984 and March 1986, was previously described [1]. The censor date of our study was set at the first day of effective antiretroviral therapy of the participant. Of the 364 participants, 131 seroconverted during the study. The remaining 233 men were positive for HIV-1 antibodies at entry between October 1984 and April 1985. In previous epidemiological studies, the time since seroconversion of these prevalent cases has been estimated based on the incidence of HIV-1 infection amongst homosexual participants of the Amsterdam Cohort and was on average 1.5 years before entry into the cohort studies [34]. For analysis, we combined the 131 participants with documented seroconversion and 233 seroprevalent participants with an imputed seroconversion date as one study group, since previous studies have not revealed differences in AIDS-free survival between the two groups [4].

The ACS has been conducted in accordance with the ethical principles set out in the declaration of Helsinki and written informed consent is obtained prior to data collection. The study was approved by the Amsterdam Medical Center institutional medical ethics committee.

### *Trim5* genotyping.

DNA samples of 327 out of 364 participants of the Amsterdam Cohort studies were available for *Trim5* genotyping. For analysis of the *Trim5* R136Q polymorphism (rs10838525), DNA samples were amplified by PCR using Taq DNA polymerase (Invitrogen) and primer pair Trim5-F (5′-ATGGCTTCTGGAATCCTGGTTAATG-3′) and Trim5-R136Q-R (5′-CCCGGGTCTCAGGTCTATCATG-3′). The following amplification cycles were used: 5min 95°C; 35 cycles of 30s 95°C, 30s 50°C, 90s 72°C; 5min 72°C. Subsequently PCR products were purified and subjected to a restriction digest with 1U Ava1 (1.5 hour 37°C; NEB) and analyzed on a 1% agarose gel. A PCR product containing an R at position 136 will result in digestion of the PCR product into a 405bp and 121bp product. A PCR product containing a Q at position 136 will result in a 526bp (undigested) product. For conformation, 15 samples (5 homozygous 136R, 5 homozygous 136Q and 5 heterozygous 136QR) have been sequenced with the ABI prism BigDue Terminator kit V1.1 (Applied Biosystems) using primers Trim5-F and Trim5-R136Q-R). Sequences were analyzed on an ABI 3130XL Genetic Analyzer.

For analysis of the *Trim5* H43Y polymorphism (rs3740996), DNA samples were amplified by PCR using Taq DNA polymerase (Invitrogen) and primer pair Trim5-F and Trim5-H43Y-R (5′-GGCTGGTAACTGATCCGGCAC-3′). For analysis of the −2GC polymorphism (rs3824949), DNA samples were amplified by PCR using Taq DNA polymerase (Invitrogen) and primer pair Tr5−2GC (5′-GCAGGGATCTGTGAACAAGAGG-3′) and Trim5-H43Y-R. The following amplification cycles were used: 5min 95°C; 35 cycles of 30s 95°C, 30s 55°C, 90s 72°C; 5min 72°C. Subsequently PCR products were purified and sequenced with the ABI prism BigDue Terminator kit V1.1 (Applied Biosystems) using primers Trim5-F and Trim5-H43Y-R for H43Y and Tr5−2GC and Trim5-H43Y-R for −2GC. Sequences were analyzed on an ABI 3130XL Genetic Analyzer.

### FACS sorting naïve and memory CD4 T cells.

Cryopreserved PBMC were stained with antibodies against CD4 (tricolor conjugated; Caltag Laboratories), CD45RO (FITC conjugated; BD Biosciences) and CD27 (phycoerythrin conjugated; Caltag Laboratories), and sorted using a MoFlo cell sorter (Cytomation Inc.). Cells were sorted in two different cell populations: naïve (CD45RO-CD27+) CD4 T cells and memory (CD45RO+) CD4 T cells.

### RNA isolation and quantitative PCR.

Total RNA was isolated from naïve and memory CD4 cells from HIV-1 infected individuals or healthy donors, using the RNeasy mini kit (Qiagen, Hilden, Germany). Subsequently, cDNA was prepared using the SuperScript™ First-Strand Synthesis System for RT-PCR (In Vitrogen). Trim5α mRNA levels were analyzed by SYBR green qPCR using the LightCycler (Roche). The reaction mix contained 20 mM Tris-HCl (pH 8.4), 50 mM KCl, 3 mM MgCl_2_, 200 μM dNTP, 250 μg/ml BSA, 500 nM primers, SYBR green I nucleic acid gel stain 40,000× diluted in water, and 0.6 U platinum Taq DNA polymerase (In Vitrogen). The following primer sets were used for detection of Trim5α cDNA: Trim5α -RNA-F 5′-ccaggatagttccttccatac-3′ and Trim5α-R 5′-agagcttggtgagcacagagtc-3′. Serial dilution of plasmid DNA containing cDNA of Trim5α were used as a standard curve. To correct for differences in the cDNA input, levels of β-actin cDNA were analyzed by a SYBR green qPCR using the following primer set: BA-RNA-F 5′-ggcccagtcctctcccaagtccac-3′ and BA-RNA-R 5′-ggtaagccctggctgcctccacc-3′. A serial dilution of 8E5 cells was used as a standard curve for β-actin. SYBR green qPCR was performed using the following program on the LightCycler: (1) preincubation and denaturation: 50°C for 2 min, 95°C for 2 min; (2) amplification and quantification: 45 cycles of 95°C for 5 sec, 55°C for 15 sec, 72°C for 15 sec; (3) melting curve: 95°C for 0 sec, 65°C for 15 min, 95°C for 0 sec with a temperature transition rate of 0.1°C/sec. Specificity of the PCR products measured using the SYBR green method was confirmed by a melting curve.

### Statistical analysis.

Kaplan Meier and Cox proportional hazard analysis were performed to study the relation between the R136Q and H43Y polymorphisms in the *Trim5* gene and disease progression. The following endpoints were considered for analysis: (1) AIDS according to the 1987 Centers for Disease Control and Prevention (CDC) definition [38]; (2) AIDS according to the 1993 CDC definition [39]; (3) CD4 T cell counts below 200 cells/μl blood; (4) viral RNA load above 10^4.5^ copies per ml blood plasma; (5) detection of X4-variants by coculture of patient PBMC and MT2 cells [37]. Fisher's exact test was used to analyze an association between the R136Q, H43Y polymorphisms and prevalence of X4-variants. Sequential Bonferroni correction (Simes-Hochberg method) was used to correct for multiple comparisons [40,41].

Univariate and multivariate relative hazards were calculated at 2 years after seroconversion for the H43Y genotype, *CCR5* genotype, presence of X4-variants at 18–30 months after seroconversion, CD4 T cells at 18–30 months after seroconversion and viral RNA load at 18–30 months after seroconversion.

Trim5α mRNA levels in naïve and memory CD4 T cells were compared using the Students *T* test.

## Abbreviations

ACS: Amsterdam Cohort Studies
CDC: Centers for Disease Control and Prevention
R5-variants: CCR5 using HIV-1 variants
RH: relative hazard
*Trim5*: tripartite interaction motif
X4-variants: CXCR4 using HIV-1 variants

## References

[ppat-0040018-b001] de Roda Husman AM, Koot M, Cornelissen M, Keet IP, Brouwer M (1997). Association between CCR5 genotype and the clinical course of HIV-1 infection. Ann Intern Med.

[ppat-0040018-b002] Smith MW, Dean M, Carrington M, Winkler C, Huttley GA (1997). Contrasting genetic influence of CCR2 and CCR5 variants on HIV-1 infection and disease progression. Hemophilia Growth and Development Study (HGDS), Multicenter AIDS Cohort Study (MACS), Multicenter Hemophilia Cohort Study (MHCS), San Francisco City Cohort (SFCC), ALIVE Study. Science.

[ppat-0040018-b003] Winkler C, Modi W, Smith MW, Nelson GW, Wu X (1998). Genetic restriction of AIDS pathogenesis by an SDF-1 chemokine gene variant. ALIVE Study, Hemophilia Growth and Development Study (HGDS), Multicenter AIDS Cohort Study (MACS), Multicenter Hemophilia Cohort Study (MHCS), San Francisco City Cohort (SFCC). Science.

[ppat-0040018-b004] van Rij RP, de Roda Husman AM, Brouwer M, Goudsmit J, Coutinho RA (1998). Role of CCR2 genotype in the clinical course of syncytium-inducing (SI) or non-SI human immunodeficiency virus type 1 infection and in the time to conversion to SI virus variants. J Infect Dis.

[ppat-0040018-b005] Kaslow RA, Carrington M, Apple R, Park L, Munoz A (1996). Influence of combinations of human major histocompatibility complex genes on the course of HIV-1 infection. Nat Med.

[ppat-0040018-b006] Klein MR, van der Burg SH, Hovenkamp E, Holwerda AM, Drijfhout JW (1998). Characterization of HLA-B57-restricted human immunodeficiency virus type 1 Gag- and RT-specific cytotoxic T lymphocyte responses. J Gen Virol.

[ppat-0040018-b007] Flores-Villanueva PO, Yunis EJ, Delgado JC, Vittinghoff E, Buchbinder S (2001). Control of HIV-1 viremia and protection from AIDS are associated with HLA-Bw4 homozygosity. Proc Natl Acad Sci U S A.

[ppat-0040018-b008] Mann DL, Garner RP, Dayhoff DE, Cao K, Fernandez-Vina MA (1998). Major histocompatibility complex genotype is associated with disease progression and virus load levels in a cohort of human immunodeficiency virus type 1-infected Caucasians and African Americans. J Infect Dis.

[ppat-0040018-b009] Stremlau M, Owens CM, Perron MJ, Kiessling M, Autissier P (2004). The cytoplasmic body component TRIM5alpha restricts HIV-1 infection in Old World monkeys. Nature.

[ppat-0040018-b010] Sayah DM, Sokolskaja E, Berthoux L, Luban J (2004). Cyclophilin A retrotransposition into TRIM5 explains owl monkey resistance to HIV-1. Nature.

[ppat-0040018-b011] Kootstra NA, Munk C, Tonnu N, Landau NR, Verma IM (2003). Abrogation of postentry restriction of HIV-1-based lentiviral vector transduction in simian cells. Proc Natl Acad Sci U S A.

[ppat-0040018-b012] Ikeda Y, Ylinen LM, Kahar-Bador M, Towers GJ (2004). Influence of gag on human immunodeficiency virus type 1 species-specific tropism. J Virol.

[ppat-0040018-b013] Hatziioannou T, Cowan S, Von Schwedler UK, Sundquist WI, Bieniasz PD (2004). Species-specific tropism determinants in the human immunodeficiency virus type 1 capsid. J Virol.

[ppat-0040018-b014] Owens CM, Song B, Perron MJ, Yang PC, Stremlau M (2004). Binding and susceptibility to postentry restriction factors in monkey cells are specified by distinct regions of the human immunodeficiency virus type 1 capsid. J Virol.

[ppat-0040018-b015] Stremlau M, Perron M, Lee M, Li Y, Song B (2006). Specific recognition and accelerated uncoating of retroviral capsids by the TRIM5alpha restriction factor. Proc Natl Acad Sci U S A.

[ppat-0040018-b016] Rits MAN, Van Dort KA, Münk C, Meijer AB, Kootstra NA (2007). Efficient transduction of simian cells by HIV-1 based lentiviral vectors that contain mutations in the capsid protein. Mol Ther.

[ppat-0040018-b017] Perez-Caballero D, Hatziioannou T, Zhang F, Cowan S, Bieniasz PD (2005). Restriction of human immunodeficiency virus type 1 by TRIM-CypA occurs with rapid kinetics and independently of cytoplasmic bodies, ubiquitin, and proteasome activity. J Virol.

[ppat-0040018-b018] Perez-Caballero D, Hatziioannou T, Yang A, Cowan S, Bieniasz PD (2005). Human tripartite motif 5alpha domains responsible for retrovirus restriction activity and specificity. J Virol.

[ppat-0040018-b019] Javanbakht H, Diaz-Griffero F, Stremlau M, Si Z, Sodroski J (2005). The contribution of RING and B-box 2 domains to retroviral restriction mediated by monkey TRIM5alpha. J Biol Chem.

[ppat-0040018-b020] Keckesova Z, Ylinen LM, Towers GJ (2004). The human and African green monkey TRIM5alpha genes encode Ref1 and Lv1 retroviral restriction factor activities. Proc Natl Acad Sci U S A.

[ppat-0040018-b021] Hatziioannou T, Perez-Caballero D, Yang A, Cowan S, Bieniasz PD (2004). Retrovirus resistance factors Ref1 and Lv1 are species-specific variants of TRIM5alpha. Proc Natl Acad Sci U S A.

[ppat-0040018-b022] Perron MJ, Stremlau M, Song B, Ulm W, Mulligan RC (2004). TRIM5alpha mediates the postentry block to N-tropic murine leukemia viruses in human cells. Proc Natl Acad Sci U S A.

[ppat-0040018-b023] Si Z, Vandegraaff N, O'Huigin C, Song B, Yuan W (2006). Evolution of a cytoplasmic tripartite motif (TRIM) protein in cows that restricts retroviral infection. Proc Natl Acad Sci U S A.

[ppat-0040018-b024] Song B, Javanbakht H, Perron M, Park DH, Stremlau M (2005). Retrovirus restriction by TRIM5alpha variants from Old World and New World primates. J Virol.

[ppat-0040018-b025] Yap MW, Nisole S, Lynch C, Stoye JP (2004). Trim5alpha protein restricts both HIV-1 and murine leukemia virus. Proc Natl Acad Sci U S A.

[ppat-0040018-b026] Ylinen LM, Keckesova Z, Wilson SJ, Ranasinghe S, Towers GJ (2005). Differential restriction of human immunodeficiency virus type 2 and simian immunodeficiency virus SIVmac by TRIM5alpha alleles. J Virol.

[ppat-0040018-b027] Kootstra NA, Navis M, Beugeling C, van Dort KA, Schuitemaker H (2007). The presence of the Trim5alpha escape mutation H87Q in the capsid of late stage HIV-1 variants is preceded by a prolonged asymptomatic infection phase. Aids.

[ppat-0040018-b028] Sawyer SL, Wu LI, Akey JM, Emerman M, Malik HS (2006). High-frequency persistence of an impaired allele of the retroviral defense gene TRIM5alpha in humans. Curr Biol.

[ppat-0040018-b029] Speelmon EC, Livingston-Rosanoff D, Li SS, Vu Q, Bui J (2006). Genetic association of the antiviral restriction factor TRIM5alpha with human immunodeficiency virus type 1 infection. J Virol.

[ppat-0040018-b030] Javanbakht H, An P, Gold B, Petersen DC, O'Huigin C (2006). Effects of human TRIM5alpha polymorphisms on antiretroviral function and susceptibility to human immunodeficiency virus infection. Virology.

[ppat-0040018-b031] Goldschmidt V, Bleiber G, May MT, Martinez R, Ortiz M (2006). Role of common human TRIM5alpha variants in HIV-1 disease progression. Retrovirology.

[ppat-0040018-b032] Nakayama EE, Carpentier W, Costagliola D, Shioda T, Iwamoto A (2007). Wild type and H43Y variant of human TRIM5alpha show similar anti-human immunodeficiency virus type 1 activity both in vivo and in vitro. Immunogenetics.

[ppat-0040018-b033] Koot M, Keet IP, Vos AH, de Goede RE, Roos MT (1993). Prognostic value of HIV-1 syncytium-inducing phenotype for rate of CD4+ cell depletion and progression to AIDS. Ann Intern Med.

[ppat-0040018-b034] Geskus RB (2000). On the inclusion of prevalent cases in HIV/AIDS natural history studies through a marker-based estimate of time since seroconversion. Stat Med.

[ppat-0040018-b035] Blaak H, van't Wout AB, Brouwer M, Hooibrink B, Hovenkamp E (2000). In vivo HIV-1 infection of CD45RA(+)CD4(+) T cells is established primarily by syncytium-inducing variants and correlates with the rate of CD4(+) T cell decline. Proc Natl Acad Sci U S A.

[ppat-0040018-b036] van Rij RP, Blaak H, Visser JA, Brouwer M, Rientsma R (2000). Differential coreceptor expression allows for independent evolution of non-syncytium-inducing and syncytium-inducing HIV-1. J Clin Invest.

[ppat-0040018-b037] Koot M, Vos AH, Keet RP, de Goede RE, Dercksen MW (1992). HIV-1 biological phenotype in long-term infected individuals evaluated with an MT-2 cocultivation assay. Aids.

[ppat-0040018-b038] Center for Disease Control. Revision of the CDC surveillance case definition for acquired immunodeficiency syndrome. MMWR.

[ppat-0040018-b039] Center for Disease Control. 1993 revised classification system for HIV infection and expanded surveillance case definition for AIDS among adolescentes and adults. MMWR.

[ppat-0040018-b040] Simes JR (1986). An improved Bonferroni procedure for multiple tests of significance. Biometrika.

[ppat-0040018-b041] Hochberg Y (1988). A sharper Bonferroni procedure for multiple tests of significance. Biometrika.

